# Supplementary material for: PPARγ controls ESCRT-dependent fibroblast-like synoviocyte exosome biogenesis and alleviates chondrocyte osteoarthritis mediated by exosomal ANXA1
Source: J Orthop Translat. 2025 Jun 27;53:187–205. doi: 10.1016/j.jot.2025.06.008 (PMC12268348; doi:10.1016/j.jot.2025.06.008)
Supplement: Multimedia component 1 [file mmc1.docx]

**
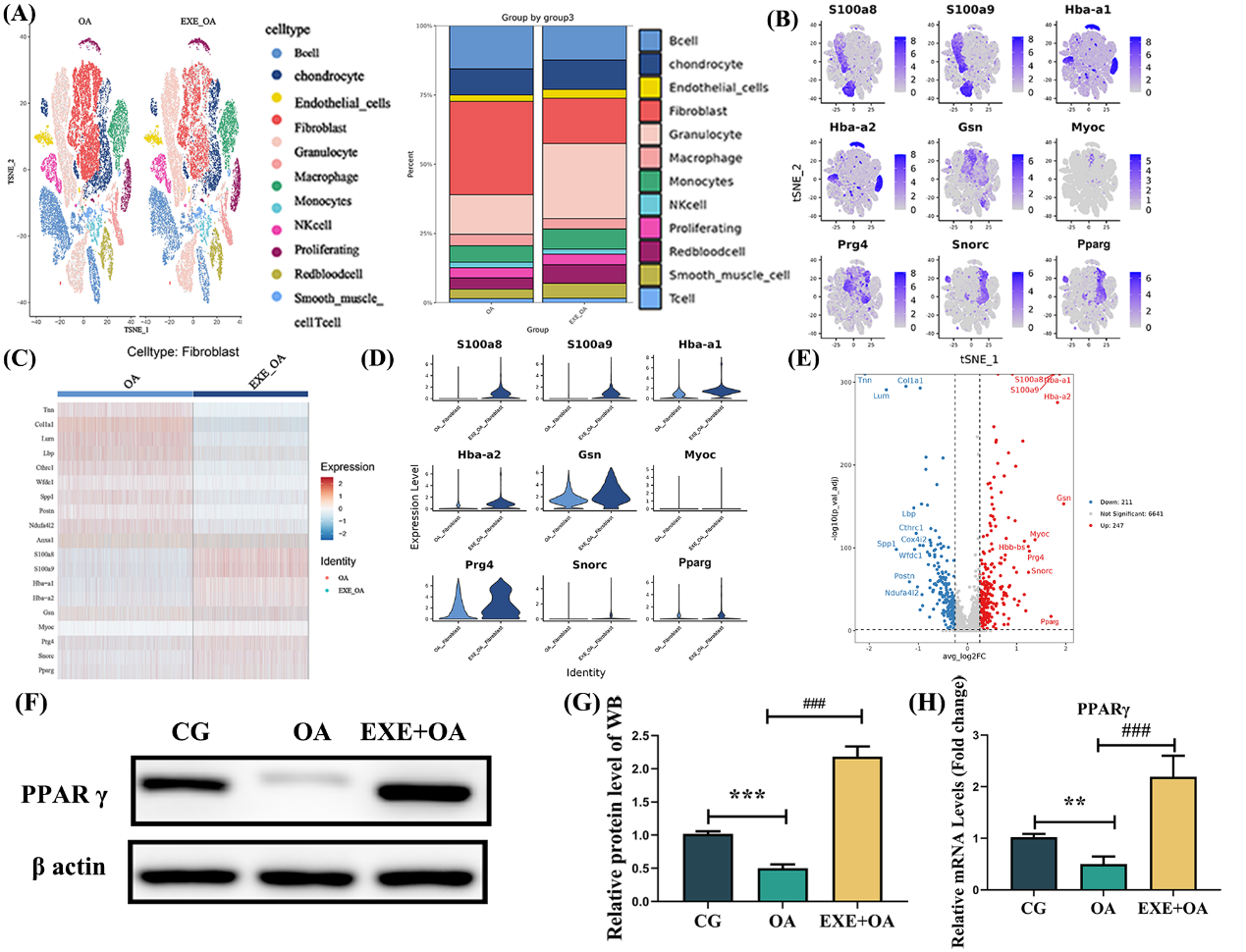
**

**Supplementary figure 1. Increased expression of PPARγ in the FLSs of the moderate exercise group is implicated in OA pathogenesis, as determined by single-cell transcriptome sequencing.** (A) A t-distributed stochastic neighbor embedding (t-SNE) map and an integrated percentage figure show the cell type distribution in the entire joint of SD rats. (B) The t-SNE map, (C) heatmap, (D) violin plot, and (E) volcano plot illustrate the top 9 upregulated genes in the FLSs when comparing the OA+exercise group versus the OA group. Among these genes, we found PPARγ to be upregulated in the FLSs of the OA+exercise group. (F) The PPARγ protein expression in chondrocytes. (G) The ratio of WB analysis. (H) The mRNA level of PPARγ detected by PCR. *p < 0.05 vs. CG; **p < 0.01 vs. CG; ***p < 0.001 vs. CG. #p < 0.05 vs. OA; ##p < 0.01 vs. OA; ###p < 0.001 vs. OA.

**
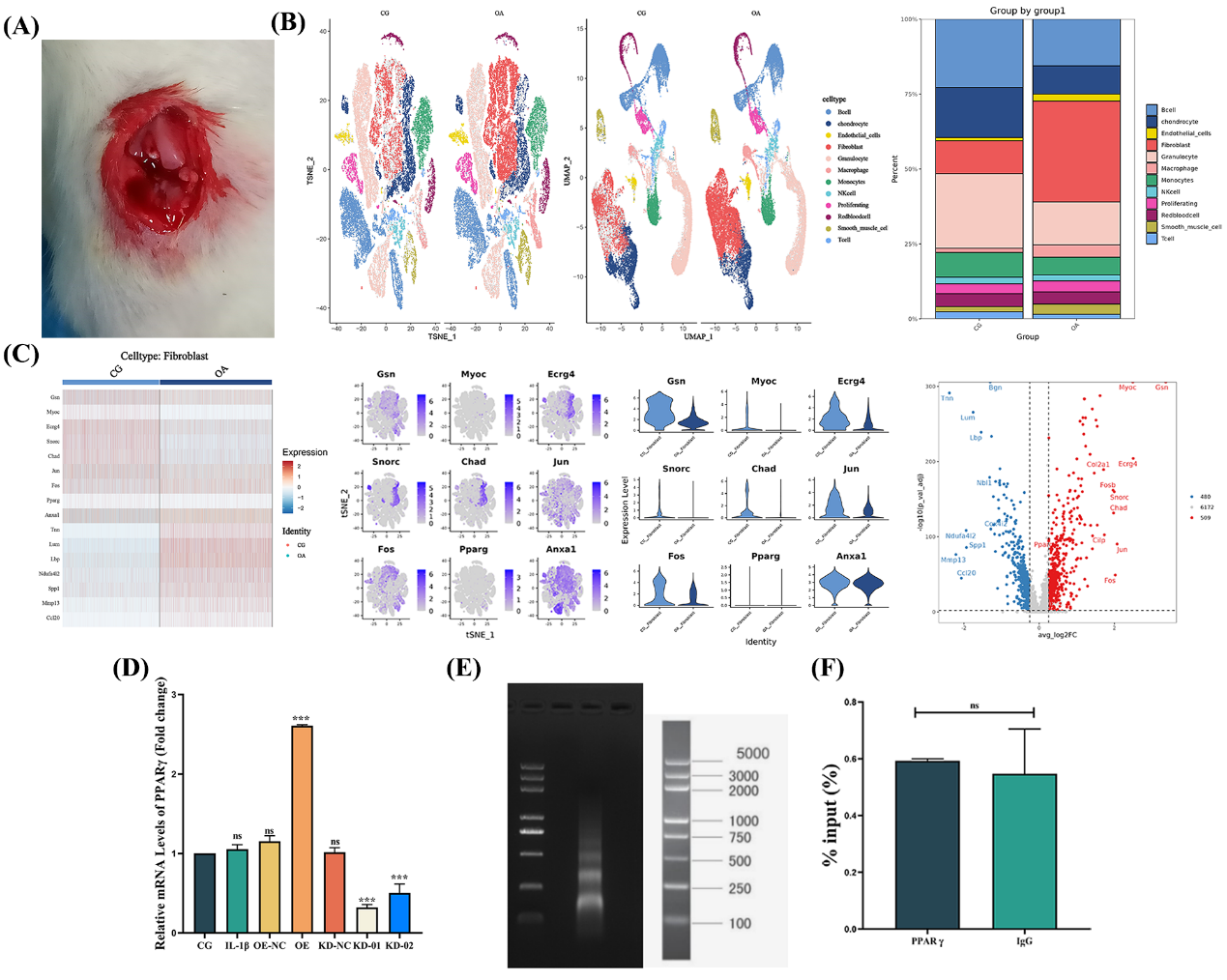
**

**Supplementary Figure 2.** (A) The ACLT osteoarthritis model. (B) t-SNE and integrated percentage figure of the cell type of the whole joint of SD rats between CG and OA group. (C) The differentially expressed gene in FLSs between CG and OA group displayed in heatmap, t-SNE, violin plot and volcano plot. (D) The relative mRNA levels of PPARγ treated FLSs to evaluate the OE or KD efficiency. (E) Results of chromatin agarose gel electrophoresis in ChIP-qPCR experiment. (F) Immunoprecipitated DNA using chromatin immunoprecipitation (ChIP) assay was quantified by qPCR in FLSs, with normal rabbit IgG as the negative control.


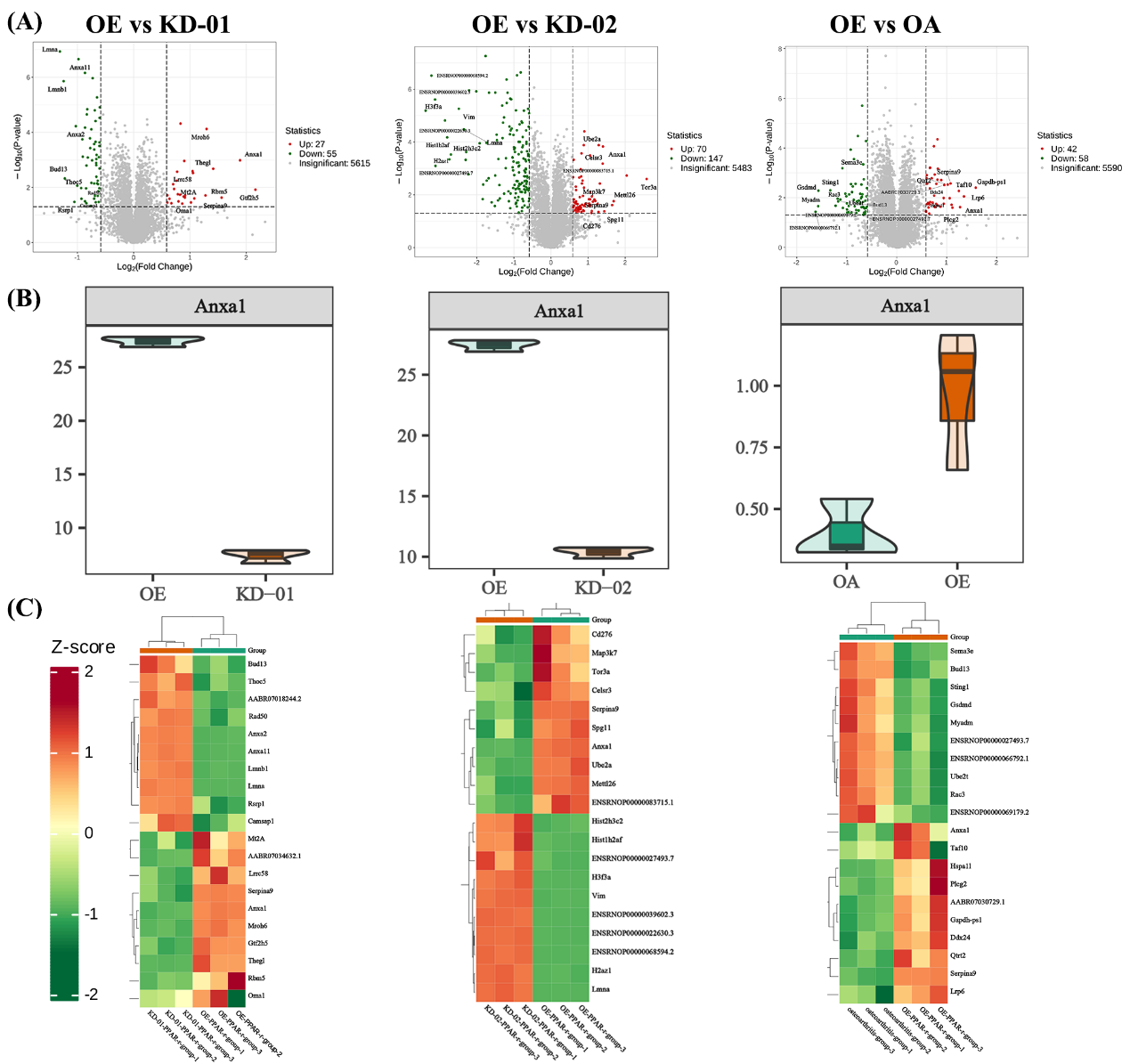


**Supplementary figure 3. Annexin A1 is enriched in exosomes derived from OE-PPARγ FLSs.** Using data-independent acquisition quantitative proteomics of FLS-derived exosomes, we found Annexin A1 to be enriched in exosomes derived from OE-PPARγ FLSs compared to KD-01, KD-02, and OA groups. The volcano plot (A), violin plot (B), and heatmap (C) depict the enrichment of ANXA1 in the OE-PPARγ group.


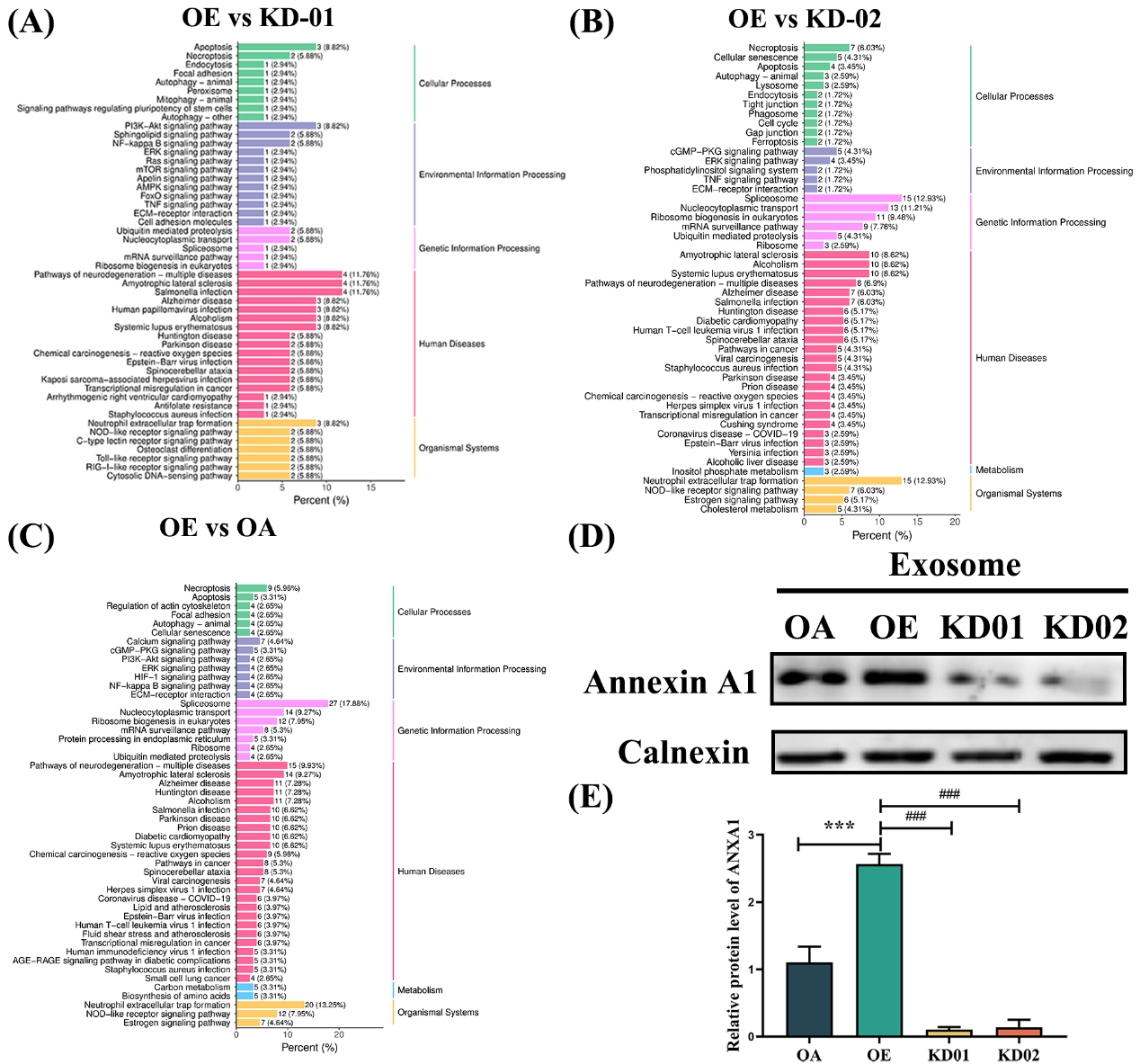


**Supplementary figure 4.** (A) (B) (C) GO analysis of OE vs KD-01, KD-02, OA group. (D) WB detection of exosomal ANXA1. (E) The ratio of WB analysis. *p < 0.05 vs. OA; **p < 0.01 vs. OA; ***p < 0.001 vs. OA. #p < 0.05 vs. OE; ##p < 0.01 vs. OE; ###p < 0.001 vs. OE.

**
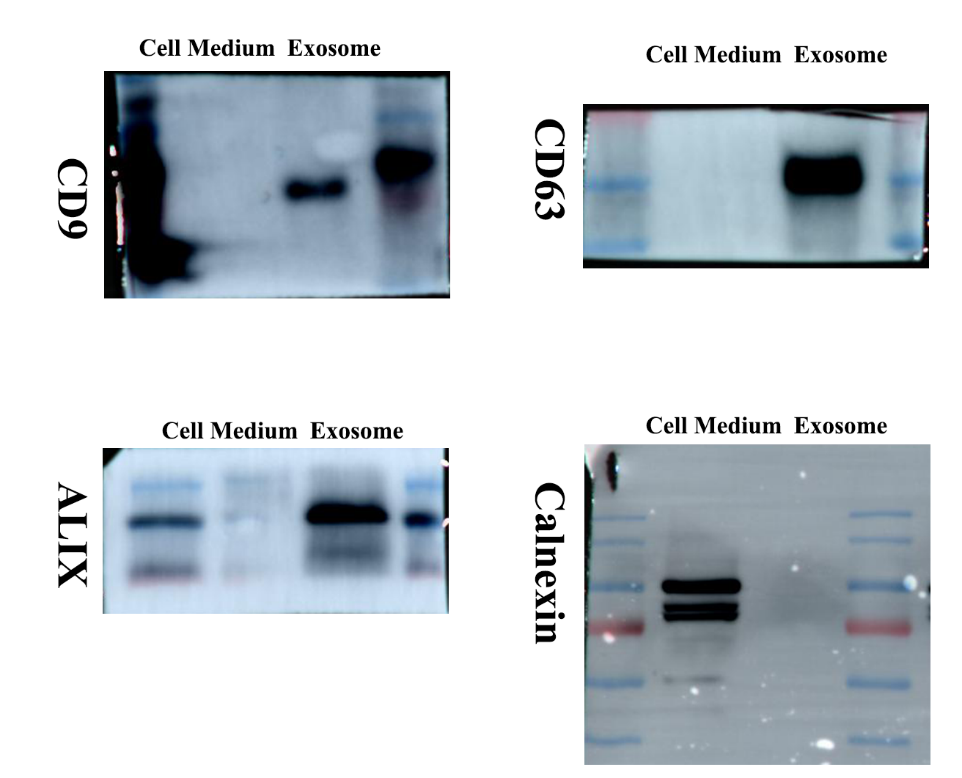
**

**Supplementary figure 5. The full WB images for exosome markers (CD9, CD63, Alix) and negative controls (Calnexin).**

**Supplementry table 1. Sequences of primers used for qRT-PCR and ChIP-qPCR**

| Target gene | Forward primer 5'-3' | Reverse primer 3'-5' |
| --- | --- | --- |
| PPARγ | TGAAGTGGAAGAGTGTTGCGTCATC | TTGGTTGGTCGGGTTTGTTTGTTTG |
| ANXA1 | GGCAGGGAGCTCAACATAGC | CCCGGAAACAACTCAGCTAC |
